# Editorial: Learning from global food and nutrition insecurity

**DOI:** 10.3389/fpubh.2024.1478523

**Published:** 2024-10-18

**Authors:** Amos Laar, Susan Vorkoper, Rafael Pérez-Escamilla

**Affiliations:** ^1^School of Public Health, University of Ghana, Accra, Ghana; ^2^Fogarty International Center, National Institutes of Health, Bethesda, MD, United States; ^3^Department of Social and Behavioral Sciences, Yale School of Public Health, New Haven, CT, United States

**Keywords:** global food insecurity, across borders, challenges, opportunities, constructs, measurement, frameworks

As the physical, economic, and epistemic access to healthy, nutritious, and safe food becomes increasingly unreliable particularly among the world's low-income groups, research on food and nutrition insecurity and dissemination of the results remains critically important both within local contexts and on the global stage. As governments worldwide become increasingly interested in developing and implementing novel and effective policy approaches to address food insecurity, it is key for researchers to help improve the stakeholders' understanding of the root causes and consequences of food and nutrition insecurity, including the inequitable distribution of the triple burden of malnutrition (1). Among others, this can be done through collaborative multidisciplinary research, evaluation and dissemination endeavors. Over the past decades, the relevant fields in this space have gained a better understanding of the epidemiology of food and nutrition insecurity within and across countries as well as its causes and consequences, although much less is known about structural policy solutions (1–3). Where evidence and knowledge exist, they tends to stay siloed and have not been extensively shared across countries and regions. This gap has led to calls for the establishment of food and nutrition and water security research networks and knowledge sharing platforms across the globe (4).

Despite the global awareness about the crucial importance of food and nutrition security for human health (1) among civil society organizations, international health agencies, and scholars, there has been little dissemination of successfully implemented and evaluated evidence-based food and nutrition security policies between countries with very few exceptions (5–7). Sharing research strategies and proven methods among researchers, policymakers, consumers and other food systems actors worldwide can help identify promising initiatives for local adaptation and implementation. For instance, by sharing experiences with evidence-informed strategies shown to be effective when properly adapted to the needs of different contexts, the world may reach a turning point to prevent and mitigate the acute and chronic food insecurity crises that have spread all over the globe. Such experiences may be shared among experts via scientific publications, expert-expert, or expert-lay people via stakeholder engagements, and government-to-people via policies.

In response to this need for broader sharing and collaboration across borders, this Research Topic emphasizes opportunities for mutual learning among researchers around the world on promising food and nutrition insecurity research. It is grounded in the Fogarty International Center's webinar series on “*Lessons learned from global food and nutrition insecurity*” conducted in the Fall of 2022. The three-part webinar series highlighted robust food and nutrition security conceptual frameworks, measurement, and indicators; innovative policy-relevant research examining the intersections of food and nutrition insecurity with other social determinants of health including water insecurity; and how these relate to poor physical and mental health outcomes worldwide. The importance of using household food insecurity experience scales for conducting research and program monitoring and evaluation was strongly highlighted as a success story of what happens when countries share their research and experiences with each other. In this instance, an initiative that started in the US rapidly disseminated globally to support regions around the world with similar efforts (5, 8).

Through this food and nutrition insecurity Research Topic, we aimed to (i) map out the food and nutrition security globally agreed upon definitions, frameworks, measurement tools, and indicators, (ii) describe the foodscapes and landscapes of global food insecurity, (iii) identify promising opportunities for implementation of effective policies and programs across different settings; and (iv) spotlight pivotal food insecurity research gaps that need to be addressed through global networks of researchers. Such sharing is needed if food insecurity and malnutrition in all its forms are to be sustainably addressed by 2030 as outlined in the United Nations Sustainability Goals.

Pérez-Escamilla sets the stage by elaborating on the food and nutrition security definitions, constructs, frameworks, and measurements of food and nutrition security, as well as applications of lessons learned at the global level. Developing and using globally agreed upon evidence-informed definitions, frameworks and measurement approaches are key given that food security is a powerful social determinant of health that is crucial for human health and planetary health (1). In this article, he makes the case for countries to benefit from the rich global experience of applying experience-based household food insecurity scales to improve our understanding across the globe of the distribution and root and more immediate causes of food and nutrition insecurity as well as its consequences for human and planetary health and for evaluating the impact of interventions designed to address it. As he points out, food security has traditionally been framed by four key dimensions, which together ensure that all people have physical, social, and economic access to sufficient, safe, and nutritious food that meets their dietary needs and food preferences for an active and healthy life—in the context of food “availability; access; utilization; and stability” (1). Recently, discussions have expanded to include the dimensions of “agency,” and “sustainability,” acknowledging their critical role in achieving food security (9, 10, 19). Including agency as a dimension of food security reflects a holistic understanding that achieving food security is not only about meeting physical needs but also about ensuring that people have the knowledge, capabilities, and freedoms to secure their dietary needs in a way that is sustainable and equitable.

Two studies focused on breastfeeding, which is a key element of the “first food systems” during the first 1,000+ days of life (11). They examined the economic benefits of breastfeeding (Smith et al.), and global lessons for strengthening breastfeeding as a key pillar of food security (Tomori). The Mothers' Milk Tool was developed to increase the visibility of the economic value that women's unpaid care work through breastfeeding infants and young children contributes to society. Smith et al. describe the development and key features of the tool, and report results for selected countries (*n* = 14) high-, middle- and low-income countries. Globally, breastfeeding women produce around 35.6 billion liters of milk annually, but 38.2% is currently “lost.” The concept of “breastmilk loss” refers to the quantity of breastmilk that is not utilized for breastfeeding despite the capacity and potential availability to do so. Such losses, Smith et al. note, are usually due to cultural and structural barriers to breastfeeding. The Mother's Milk tool is valuable to food and health policymakers, advocates, researchers, and individual mothers by attributing a monetary value to breastmilk production. It shows what is at risk economically for nations and the world if women's capacity for breastfeeding is not protected, promoted, and supported by effective national policies, programs, and investments. Tomori presents the global lessons for strengthening breastfeeding as a key pillar of food security. This paper highlights the central importance of breastfeeding for food security across diverse global settings by examining three case studies from Honduras, Pakistan, and the USA. Lessons drawn from these case studies (including low prioritizing of breastfeeding and suboptimal incorporation of infant and young child feeding protocols into disaster preparedness into the policy agenda, as well as ensuring that first food security is considered in energy policy) reinforce the importance of multisectoral collaboration to scale up investment in creating equitable, enabling environments for breastfeeding. An integrated approach to policy change is necessary to recognize and strengthen breastfeeding as a pivotal part of ensuring food security across the globe (Tomori).

Two other studies address food insecurity among adolescents. Sridhar et al. responds to the many global calls not to leave the adolescents behind in the global fight against food insecurity and malnutrition. Adolescents make up roughly a quarter of the population in Zambia; however, most food and nutrition security-related programming is targeted at children under 5 years old. Their work shows that the prevalence of malnutrition in adolescents and older children living in a Zambian district was comparable to those under five calling for interventions that address both age groups. In a separate study, Osei Bonsu et al. used data from the global in-school students survey to examine the relationship between food insecurity and sleep disturbance among almost 200,000 school going adolescents They concluded that reducing food insecurity could be an effective policy strategy for enhancing adolescent sleep quality and thus overall quality of life. A youth-focused study by Mokari-Yamchi et al. utilized data from the US National Health and Nutrition Examination Survey 2017–2018 to examine the prevalence of household food insecurity in connection with specific sociodemographic factors and its association with obesity. Their analysis revealed that youth from food insecure households were more likely to be obese (adjusted odds ratio [aOR]: 1.59, 95% confidence interval [CI]: 1.19–2.13) and to have abdominal obesity (aOR: 1.56, 95% CI: 1.19–2.03). In contrast, factors such as having a head of household with a college degree and a household income exceeding 350% of the poverty line were associated with a reduced risk of experiencing household food insecurity.

Focusing on two sub-Saharan Africa countries—Ethiopia and Tanzania—two studies examined different dimensions of food security including food availability in Ethiopia (Wubetie et al.), agency in rural Tanzania (Madzorera et al.). Wubetie et al. examined the levels of household food insecurity in Ethiopia considering geographic, environmental, and socioeconomic variables and compared this to measurements of food insecurity in Ethiopia using the United Nations World Food Program's Food Consumption Score. They conclude that the -recommended cut off points in WFP consumption score underestimated the prevalence of household food insecurity, which has both policy and programmatic implications. Madzorera et al. evaluated the associations of women's participation and decision-making in key agricultural and household activities with women's diet quality. They found that women's input and decision-making in agriculture were associated with improved diet quality in rural Tanzania.

Martinez-Brockman et al. examined the risk factors for household food insecurity using data from the Eastern Caribbean Health Outcomes Research Network Cohort (ECHORN). They showed that demographic, psychosocial, behavioral, and environmental risk factors were associated with household food insecurity among adults 40 years of age or older in the ECHORN cohort. In contrast to previous studies, the researchers did not find that women in the cohort had a higher risk of household food insecurity compared to men, although a different set of risk factors affected men and their vulnerability to household food insecurity. This underscores the complexity and multidimensionality of how different factors affect household food insecurity across different contexts.

Gaitán-Rossi et al. examined the persistence of household food insecurity in the context of the COVID-19 pandemic in Mexico using machine learning to identify predictors of persistent moderate or severe household food insecurity. They found that the most consistent and influential predictors of household food insecurity were household food insecurity at the beginning of the study period, lower socioeconomic status, not being able to adopt financial coping strategies, and not receiving government support. The authors suggest that governments should consider these factors for identifying households that may be less responsive to food insecurity policies when prioritizing government support.

Two papers in this Research Topic looked at the implications of external shocks on food insecurity. Bangham et al. examined the effect of adverse economic events (including job loss, changes in family structure, and poor health) on hunger severity among food pantry clients in Boston, USA. Their data show that unexpected or increased medical expenses, job loss, pay reduction, and the death of a family member were associated with moderate to severe hunger. They concluded that anticipating the impact of adverse economic events on food insecurity can inform preparedness for public health programs and policies for people in need of additional resources, which is essential for their wellbeing in times of increased economic instability. Using a case study approach, Dietz and Fanzo explored the bidirectional relationship of the U.S. agrifood sector to climate change. For instance, cattle production for beef consumption generates methane and nitrous oxide, both of which are potent greenhouse gases. These gases contribute to global warming which in turn increases the frequency and strength of adverse catastrophic events, which compromise the food supply. Increased greenhouse gases also affect crop yields and the micronutrient content of crops, which adversely affect the prevalence of food and nutrition insecurity, particularly in low- and middle-income countries. Such complexities impact the ability to develop sustainable food systems and call for meaningful and sufficient engagements of key food systems actors, emphasizing the critical need to build the political will for change. Both articles clearly illustrate the need for local solutions to collectively address a global existential problem and the engagement of the community through knowledge sharing platforms and robust action-oriented evidence-informed networks.

Finally, two studies examined “produce prescription” programs that are part of the US “Food is Medicine” initiative. Broadly, food as medicine interventions address food insecurity among patients and at the same time deploy nutrition-based interventions many of them targeting chronic diseases (12–14). Among others, these programs improve food security by increasing access to fresh fruit and vegetable consumption, by giving money to their clients earmarked for purchasing fresh produce in local supermarkets and other food retailers. Owens et al. evaluated a hospital-based food and nutrition programming. The program was delivered by a level 1 trauma health care system in Atlanta, Georgia, USA in partnership with community-based organizations. They found that the Food as Medicine program provides a novel model for building health equity through food within healthcare organizations. They concluded that the intervention was feasible but required further improvements for further successful scale up or transferability toward improving food security and human wellbeing for patients nationwide. Clients' experiences and satisfaction with “produce prescription” programs targeting low-income people in California were examined in a rich qualitative study by Rhodes et al.. While evidence shows that produce prescription programs can improve food security, fruit, and vegetable consumption, and ultimately health outcomes, clients' satisfaction with the programs is critical. Clients' experiences and satisfaction with “produce prescription” programs targeting low-income people in California were examined in a rich qualitative study by Rhodes et al.. While evidence shows that produce prescription programs can improve food security, fruit, and vegetable consumption, and ultimately health outcomes, clients' satisfaction with the programs is critical. Rhodes et al. reported that clients were quite satisfied with the program but at the same time offered recommendations on how to ensure that the programs services are delivered with dignity and respect to all clients. Their findings inform efforts to make “produce prescription” programs more person-centered and respectful, which in turn may increase program demand, engagement, and impact. As other countries conduct or consider initiating similar prescription programs, the evidence provided in this article could help inform the program co-design and implementation approaches.

The series of articles in this Research Topic represent some of the global nutrition and food insecurity research happening across the lifespan and explores innovative research interventions and methodologies on food insecurity that is of critical importance both domestically and abroad highlighting opportunities for mutual learning. As an example, the paper by Smith et al. (15) was recently cited in the Bulletin of the World Health Organization in an article detailing why evidence-based breastfeeding protection, promotion, and support should be officially incorporated by governments all over the world as a carbon offset intervention to mitigate climate change. It is our hope that this Research Topic can inform efforts to find better ways to improve food and nutrition security governance (5), policies, and programs around the world, particularly in areas where people are disproportionately affected by food insecurity as this condition has wide-ranging short- and long-term physical and mental health consequences.

To conclude, it is apropos to note the need to address the ongoing debates surrounding definitions and frameworks of global food and nutrition insecurity, which are often shaped by the perspectives of the Global North. These definitions can reflect cultural values that may not resonate with or apply to the diverse realities of those living in the Global South. Additionally, the dynamics of global trade play a significant role in shaping food insecurity. Power relations inherent in trade agreements and policies often exacerbate vulnerabilities for low-income groups, both in the Global South and within marginalized communities in the Global North.

Moving forward therefore, it is essential to fully incorporate the viewpoints of those affected by food insecurity, particularly people in the Global South and low-income populations in the Global North. This balanced approach will enhance our understanding of food insecurity and inform the development of equitable, well-coordinated policies across social, economic, agricultural, and healthcare sectors (16–19). Addressing the power imbalances in global trade is vital, as these imbalances significantly impact social determinants of health inequities, including food insecurity, within unhealthy and unsustainable food systems (1).
